# Supplementary material for: Individual pupil size changes as a robust indicator of cognitive familiarity differences
Source: PLoS One. 2022 Jan 21;17(1):e0262753. doi: 10.1371/journal.pone.0262753 (PMC8782349; doi:10.1371/journal.pone.0262753)
Supplement: S1 Fig — a-c) Bend correlations of median scores on the brand familiarity scale with the brand knowledge, brand experience, and product use scales, respectively. Median scores of the 300 images that were used in the pupillometry study plotted. This is a subset of the median scores of all images obtained through the validation survey depicted in Fig 1a. Correlation coefficients and p values obtained from robust bend correlations [37]. Solid black lines depict best fit lines. Circles denote values downweighted in the x, y, and both dimensions (red, green, and black, respectively; 5% of values). (PDF) [file pone.0262753.s001.pdf]

Supporting Information

Individual pupil size changes as a robust indicator of cognitive familiarity differences

Léon Franzen[1,2,3¶*], Amanda Cabugao[1¶], Bianca Grohmann[2], Karine Elalouf[1], Aaron P. Johnson[1,4]

[1] Department of Psychology, Concordia University, Montréal, Quebec, Canada.

[2] Department of Marketing, John Molson School of Business, Concordia University, Montréal, Quebec, Canada.

[3] Department of Psychology, University of Lübeck, Lübeck, Schleswig-Holstein, Germany.

[4] Vision Health Research Network, Montréal, Quebec, Canada.

¶ These authors contributed equally to this work

* Corresponding author

E-mail: leon.franzen@mail.com (LF)

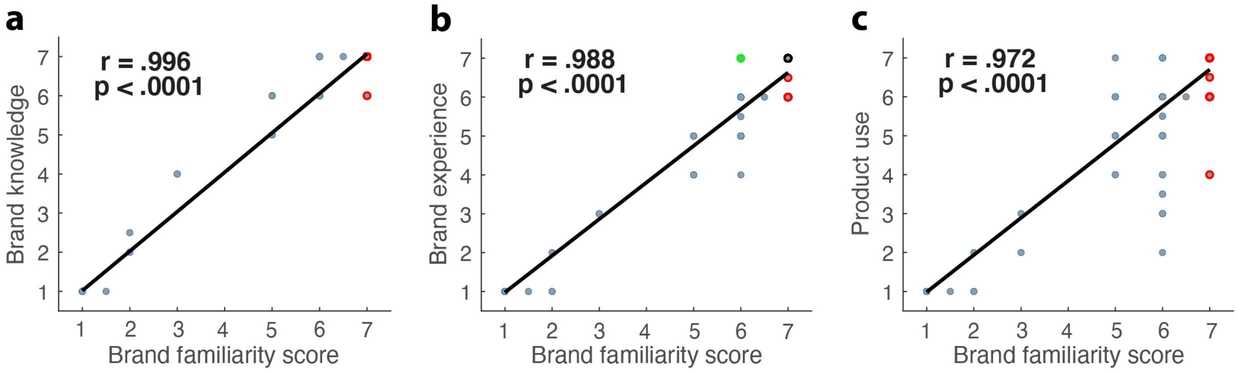

S1 Fig. Correlation of validation scores of the experimental stimulus set.

Data availability

All data files supporting the results are available from the project's Open Science Framework repository (https://osf.io/3w5s6/).
