# Supplementary material for: Individual pupil size changes as a robust indicator of cognitive familiarity differences
Source: PLoS One. 2022 Jan 21;17(1):e0262753. doi: 10.1371/journal.pone.0262753 (PMC8782349; doi:10.1371/journal.pone.0262753)
Supplement: S2 Fig — a) Individual difference between familiarity conditions relative to baseline and individual dynamic range by product category in percent of an individual’s dynamic range. Dashed grey lines depict the individual mean baseline across all trials of this participant. Colorful solid lines illustrate the effective pupil size difference. Product categories are depicted as follows: Beverages (green), personal care (purple), cleaning (red), and food (orange). b) Fraction of participants showing an effect similar to the direction of the entire group for each product category. Results computed for each window. Dashed grey lines depict 50% of participant showing the same effect as the group. Color scheme as in panel a. (PDF) [file pone.0262753.s002.pdf]

Supporting Information

Individual pupil size changes as a robust indicator of cognitive familiarity differences

Léon Franzen[1,2,3¶*], Amanda Cabugao[1¶], Bianca Grohmann[2], Karine Elalouf[1], Aaron P. Johnson[1,4]

[1] Department of Psychology, Concordia University, Montréal, Quebec, Canada.

[2] Department of Marketing, John Molson School of Business, Concordia University, Montréal, Quebec, Canada.

[3] Department of Psychology, University of Lübeck, Lübeck, Schleswig-Holstein, Germany.

[4] Vision Health Research Network, Montréal, Quebec, Canada.

¶ These authors contributed equally to this work

* Corresponding author

E-mail: leon.franzen@mail.com (LF)

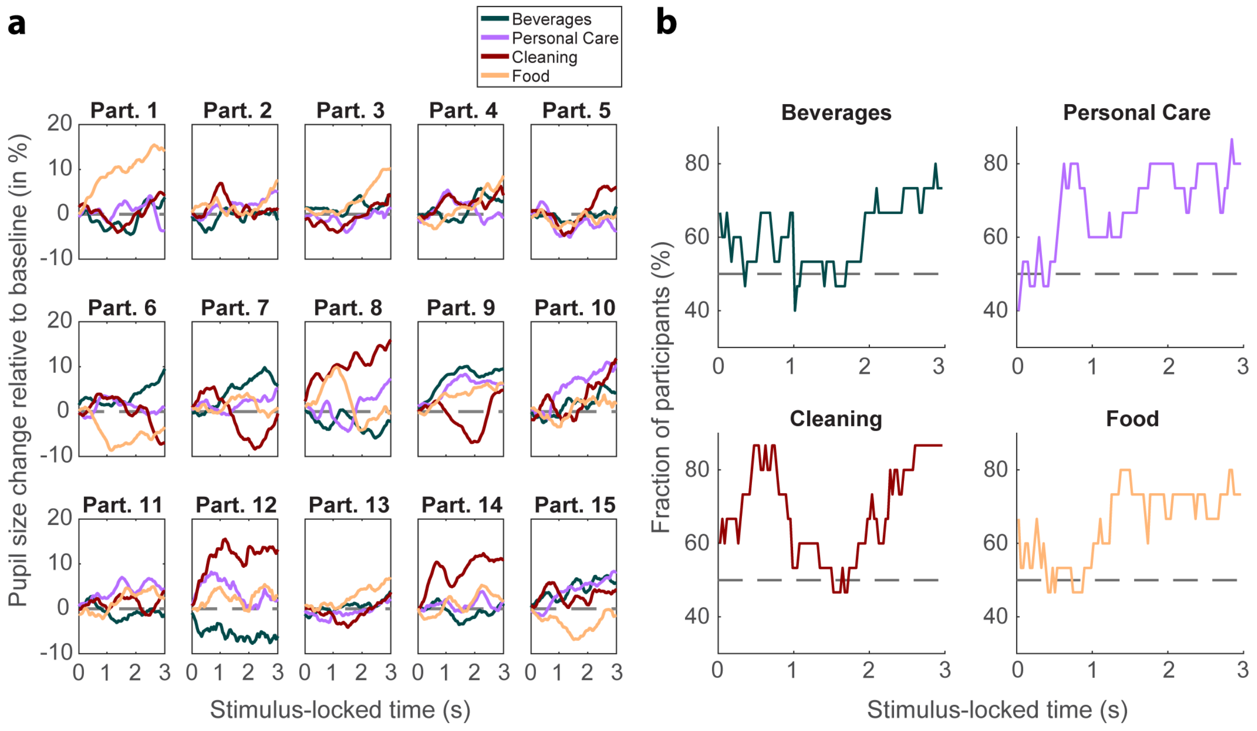

S2 Fig. Pupil size change relative to baseline and individual dynamic range by product category.
