# Supplementary material for: Individual pupil size changes as a robust indicator of cognitive familiarity differences
Source: PLoS One. 2022 Jan 21;17(1):e0262753. doi: 10.1371/journal.pone.0262753 (PMC8782349; doi:10.1371/journal.pone.0262753)
Supplement: Attachment — Submitted filename: Response to Reviewers_Revision2_FranzenEtAl.pdf [file pone.0262753.s004.pdf]

We thank the reviewers for their very constructive feedback in the last round of reviews. We have aimed to address all their comments and concerns in the revised manuscript, as detailed in the point-by-point response below (see sections in blue font). Where we took explicit action is indicated by the word ACTION. Line numbers refer to the revised manuscript without tracked changes.

Comments to the Author

Reviewer #1: All comments and concerns were addressed. The manuscript is now in my opinion technically sound, with data that support the conclusions and rigorous statistical analyses. Underlying data have been made availalble and the manuscript is in standard english.

We would like to thank this reviewer for their constructive comments in the process and are pleased to hear that all concerns have been addressed satisfactorily.

Reviewer #2: The authors have addressed all my comments. However, my main concerns regarding phase-scrambling as a better baseline and the question how feasible pupillometry is as an application in market reserach remain. Also, I don't think that the plotting of fixation points helps to check for a potential confound of gaze angle (larger gaze angles lead to smaller pupil sizes) and thus whether the pupil differences between the familiar and unfamiliar conditions are confounded by the radius of gaze angle (see point 4 of reviewer 1).

This comment is surprising to us for two reasons. First reviewer 1, who brought up this issue in the first place, was satisfied with the additional analysis we carried out as part of the last round of reviews. In this analysis, we demonstrated that no larger gaze angles were present that should have been a reason for concern. While we agree with the assertion that larger gaze angles can lead to smaller pupil sizes and that we did not directly examine pupil size as a function of gaze distance to the center of the screen, this confound should be ruled out by the absence of such larger gaze angles. To directly address the remaining concern in the present round of reviews, we have carried out additional analyses quantifying the average gaze distance to the center of the screen for each familiarity condition and (2) calculated the average pupil size by binned gaze position (Fig. 3). Both analyses demonstrate that there is no reason for any concern. Please see our responses towards the end of this document for more details.

First, I do not agree with the authors argumentation why phase-scrambling is problematic. Oliva & Torralba's results refer to a computer vision algorithm trained to categorize natural scene images converted to the phase domain. This paradigm and task is totally different from humans categorizing phase-scrambled images, which is really really hard, if not impossible with 0% phase coherence. Zero percent phase coherence also gets rid of any clues about contour. That phase-scrambling is a relatively advanced technique, is also not a good reason not to choose it. I understand it is out of the scope of the study to redo the experiment with better baseline images. I only expected that the authors at least consider phase-scrambling as an improved baseline paradigm to be described in the discussion. Because the authors have removed any reference to their baseline method as novel and innovative, the current, rather simple baseline implementation isn't a major issue anymore.

We would like to clarify/re-iterate that we are *not* saying familiarity is recognizable and discriminable from phase-scrambled images themselves—particularly not those at 0% phase coherence. However, phase scrambling works better for grey-scaled images, as it results in

a scramble of somewhat homogenous luminance across the entire image. Fig. 1, which we also provided during the last round of reviews, illustrates this point. It does not appear to work equally well for bright and colorful RGB images. Please see below, for examples of a variety of product packages from the present study (Fig. 2). We acknowledge that our point could have been made clearer by applying phase scrambling to (the original) colored product images in the last round of reviews. Applying phase scrambling to colored RGB product images, it appears that phase scrambling achieves worse results for the purpose of our study. When applied to these brightly colored images, a phase-scrambled baseline could (1) suffer from larger high luminance patches within the image and (2) simply give away more clues about the image (i.e., arrangement of colors, potential size, and product shape) than the pixel-scrambled images that we used in the present study do. If the visible high luminance patches are fixated during the baseline period, this could substantially drive pupil dilation/constriction, whereby it would counteract the baseline's goal of adapting the pupil to the average luminance of the image, while giving away as little cues about the upcoming product as possible. Achieving these goals seems to be more likely with each pixel neighboring another random pixel from the image.

A reason for these differences between grey and colored images is that more high frequency vertical artifacts remain when applying phase scrambling to colored images (i.e., to each RGB channel). These artifacts appear to give away more information about the product than the pixel-scrambled image would, even at 0% phase coherence. Specifically, information about some edgy/squary products could happen to be retained (Fig. 2 top row), while rounder products are being rendered unnoticeable, indeed. The shape of the to be expected object would then matter more for some product categories that have rather uniform packaging with strong edges.

Hence, we argue that the pixel-scramble baseline method might be equally effective, if not even advantageous, depending on the stimulus set, and that its simplicity should be considered a virtue in the context of bridging psychophysics and consumer psychology research.

ACTION: In the revised manuscript, we discuss the phase scrambling technique further. In this discussion, we emphasize that phase scrambling is an alternative technique that could lead to the same efficient adaptation of the pupil to an image's overall properties. However, since we are not aware of an empirical investigation of phase-scrambled product images for the purpose of a familiarity classification by pupil size, we are not comfortable with making a clear statement on which of the two techniques is better suited. This question will have to be answered based on actual data from future empirical studies. (Lines 692-693 & 700-706)

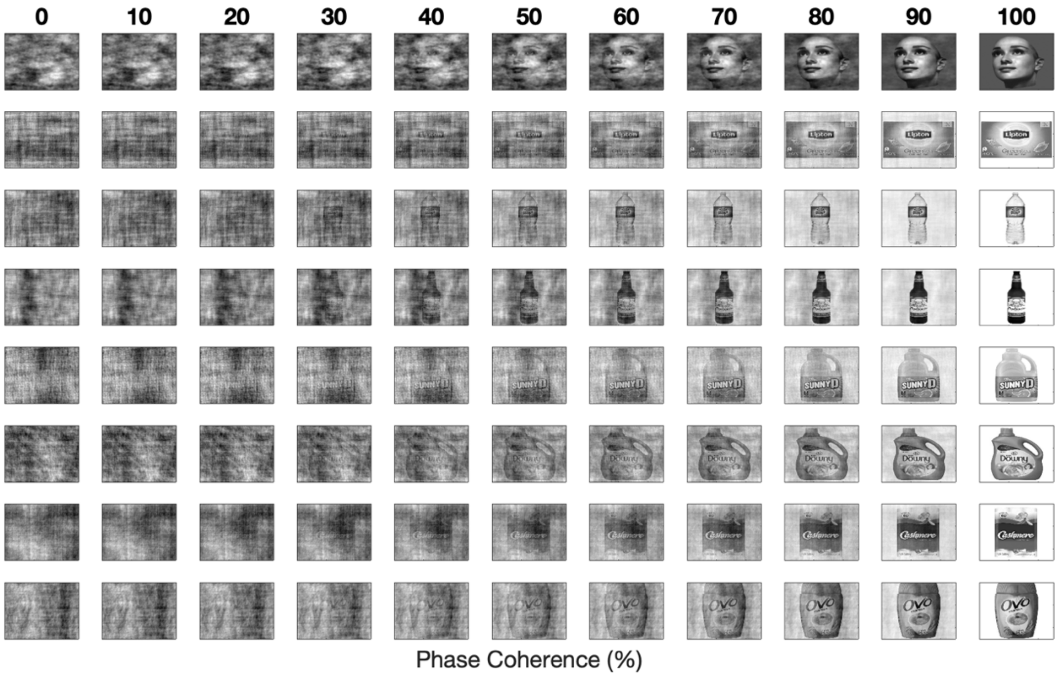

Figure 1. Examples of grey-scaled phase-scrambled face and product images at increasing levels of phase coherence (phase coherence increases from left to right).

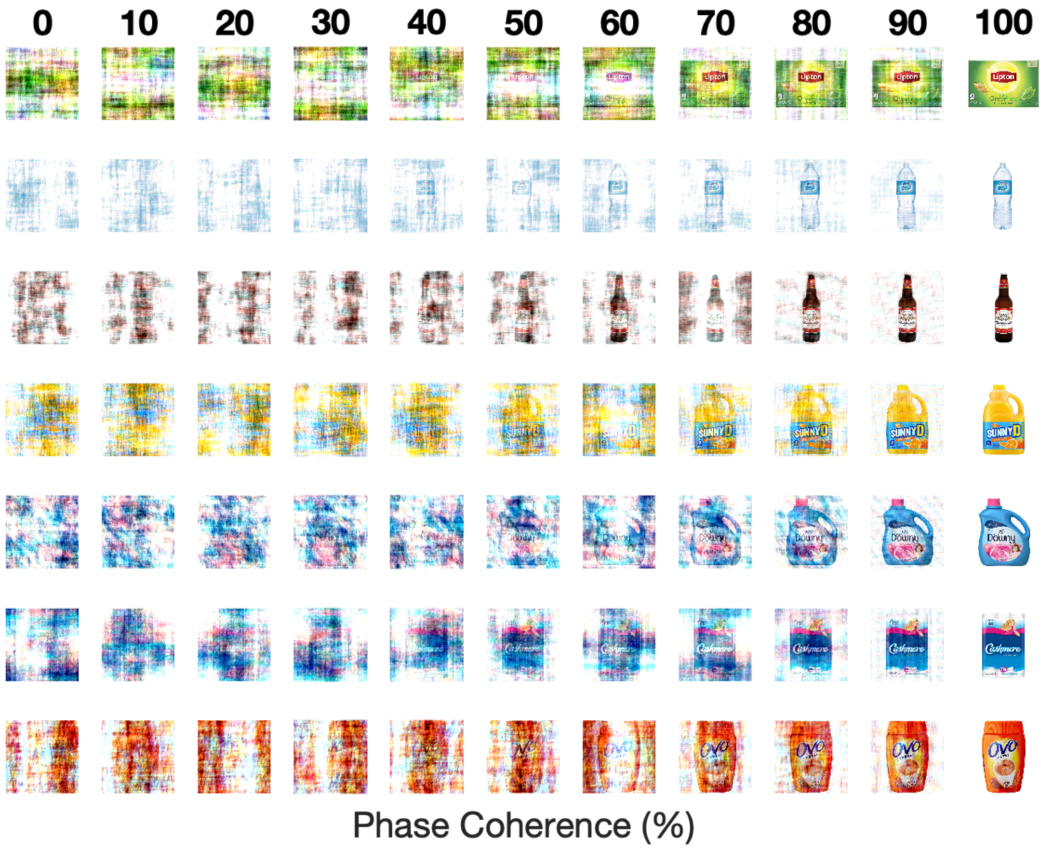

Figure 2. Examples of phase-scrambled original RGB product images from the present study at increasing levels of phase coherence (phase coherence increases from left to right).

Second, please correct me if I am wrong, but I understand from the new discriminant analysis that familiar versus unfamiliar images can be classified based on pupil size merely above chance performance (0.52>0.50). This low score basically suggests that pupillometry is not a feasible technique to determine a person's image familiarity and that it is much more efficient to just ask them how familiar observers are with the images. This should be discussed. Although this finding reduces the impact and novelty of the findings, I still appreciate the effort the authors have put in this experiment and paper, and because the paper generalizes previous findings to an applied setting, I think the paper remains relevant to the field of pupillometry and market research.

We agree with our paper's relevance for the field. Here, we would like to take the opportunity to comment on the seemingly small gap between chance and our classification performance.

First, although a classification value of 0.52 is close to chance level, two points are important to keep in mind when interpreting this result. 1) Due to the singularity of the pupil signal, only information from one channel was available for this multivariate discriminant analysis. In general, this discriminant analysis is rather conservative and dependent on the self-similarity of an experimental condition (i.e., product images) and its answers. It often yields discrimination performance of "only" around 0.6 after a single exposure to the stimulus material when applied to 64 EEG channels in established face research (Franzen et al., 2020; Diaz et al., 2017). We have seen this type of analysis fail altogether (i.e., only providing chance level discrimination performance) even when data from 64 or more EEG channels was used that otherwise led to large and significant ERP differences, which survived rigorous statistical testing. Hence, the provided discriminant analysis simply shows that a certain level of discrimination ability is already possible from pupillometry data alone in response to a large variety of product images, as used in the present stimulus set. Nevertheless, we acknowledge that this level is not much above chance for the present stimulus set.

Second, we did not intend to say pupillometry alone is absolutely and always sufficient to categorize a wide variety of product images in familiar and unfamiliar categories reliably on a single-trial basis in a variety of settings. Rather, and as explicitly stated in the guidelines added in the last round of revisions, it may be the complementary nature of pupillometry and carefully selected self-reports that seems to be most promising for future applied market research. As far as our data goes, we argue that we demonstrate that pupillometry is a suitable complement to the traditionally used and very prevalent reliance on self-reports. It constitutes another non-intrusive psychophysiological tool for providing additional time-resolved information that would not be available by self-reports alone. Importantly, pupillometry allows researchers to look at the evolution of this signal over time, which self-reports are incapable of. Lastly, above chance classification accuracy might be gathered from more constrained stimulus sets and of value in settings where no active responses could be obtained from the participant via self-reports or button presses.

ACTION: We clarified this point on the potential impact of pupillometry alone in market research in the discussion of the revised manuscript. Our focus lies on arguing that pupillometry could be used more frequently in the future, while still benefitting from being complemented with a few carefully selected self-report measures. (Lines 730-733)

Third, I would like to respond to the reply to a point made by the other reviewer about how optical distortion due to the difference between gaze and eye-tracker angle on pupil size. I can imagine why the authors do not want to invest a lot of time in implementing a model and controlling for a potential confound, although personally I would try to at least get more insights into the potential presence of this confound, if not only to satisfy the reviewer and show appreciation for their feedback.

I suggest that the authors calculate the mean gaze angle deviation from the center of the screen (i.e., average fixation radius; mean(sqrt((x_gaze-width_screen/2)^2 + y_gaze-height_screen/2)^2)) per familiarity condition and see whether this differs.

As mentioned earlier, we consider pointing out our response to the other reviewer as insufficient—who was fully satisfied by our response—an interesting practice of scientific rigor. Regardless, since the point has merit, we have carried out the suggested control analysis using two separate robust measures of central tendency to compute our averages, namely the median and 5% trimmed means. Results of both measures show virtually identical average deviations from the center of the screen for both familiarity conditions ($Median_{all}$ = 69.25, $Median_{familiar}$ = 70, $Median_{unfamiliar}$ = 68.64; 5% trimmed mean: M_{all} = 77.41, $M_{familiar}$ = 77.69, $M_{unfamiliar}$ = 77.12; in pixels). These results clearly demonstrate that the average deviation from the center should not have introduced a confound.

ACTION: We have added these results to the results section of the revised manuscript (lines 428-432).

As a second control analysis, the authors should plot pupil size as a function of horizontal and vertical gaze position. They can bin gaze positions across the screen locations and plot the average pupil size per bin.

If any differences in pupil size are observed (between familiar and unfamiliar, and across fixated screen locations), I suggest to incorporate gaze radius, and/or horizontal and vertical gaze positions as nonlinear, covariates in the discriminant analysis to see how much it explains away the difference in pupil size between the conditions.

We consider the chance of our data being subject to this EyeLink-specific issue unlikely due to the rather small size of the used images and the actual products within them. Almost all fixations and saccades occurred around the center of the display, as our fixation plots visually demonstrated in the last round of reviews. Nevertheless, since our last response only showed qualitative evidence of most fixations occurring in the center of the screen, we would like to provide more quantitative evidence showing the same result here (Fig. 3). As suggested, we re-analyzed our data and plotted the average (median & 5% trimmed mean) pupil size per X-Y bin of 32 pixels.

The results of this control analysis demonstrate that most fixations occurred close to the center of the screen (Fig. 3b, e, h). More importantly, the average baseline corrected pupil size is similar within a large circle around the center of the display (Fig. 3a, d, g). A few bins towards the edges of the display show higher pupil size averages (darker colors; Fig. 3a, d, g), however, these bins only comprise less than 20, if not even fewer, samples across the entire study (Fig. 3c, f, i). Such low numbers do not have the power to confound our results in any meaningful way, as their number is insignificant, and we have been using robust measures of central tendency throughout this entire study. These differences are also not systematic. In addition, the slight differences in arbitrary units towards locations away from the center of the screen, which we observe here, is common across standard eye-trackers.

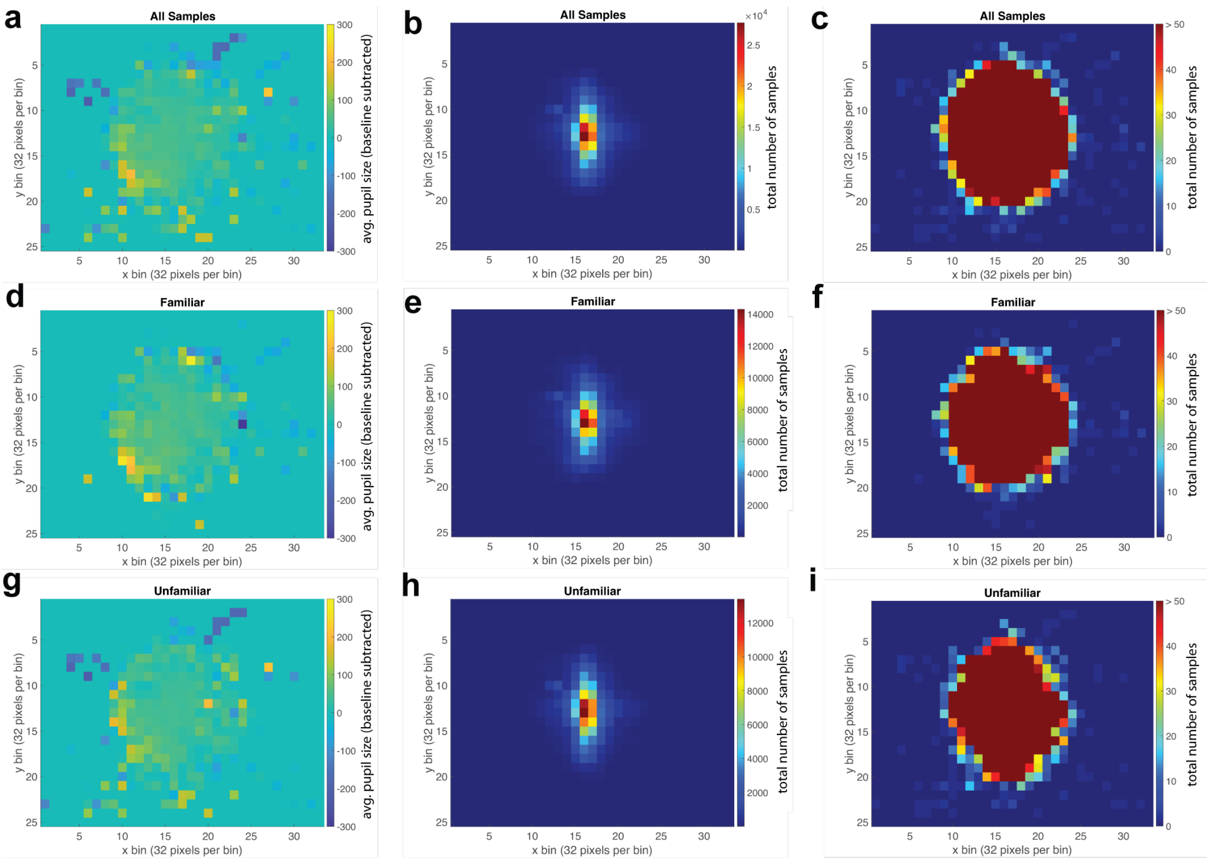

Figure 3. All panels represent the screen and its binned locations. **a, d, g)** Average pupil size (i.e., 5% trimmed mean) in arbitrary units after subtractive baseline correction as a function of X and Y screen coordinate bins. Bins comprise of 32 pixels. Blue-green color towards the edges depicts zero or no available values. **b, e, h)** Total number of samples in each bin across (b) the entire study, (e) familiar trials, (h) unfamiliar trials as a function of X and Y screen coordinate bins. **c, f, i)** Total number of samples using a different colorbar scaling with the maximum (dark red) depicting bins with more than 50 samples. These panels show that large values in panels (a, d, g) are a result of fewer than 10 samples (dark blue).
