# Supplementary material for: Individual pupil size changes as a robust indicator of cognitive familiarity differences
Source: PLoS One. 2022 Jan 21;17(1):e0262753. doi: 10.1371/journal.pone.0262753 (PMC8782349; doi:10.1371/journal.pone.0262753)
Supplement: Attachment — Submitted filename: Response to Reviewers_FranzenEtAl.docx [file pone.0262753.s003.docx]

We thank the reviewers for their very constructive feedback. We have aimed to address all their comments and concerns in the revised manuscript, as detailed in the point-by-point replies below (see sections in blue font). Where we took explicit action is indicated by the word ACTION. Sections written in Times New Roman font are direct quotes from the revised manuscript. Line numbers refer to the revised manuscript without tracked changes in its compiled pdf format, not the underlying word document. On the last page of this response document, we provide a list of the references that we added as part of our arguments.

Reviewers' comments:

Reviewer's Responses to Questions

**Comments to the Author**

1. Is the manuscript technically sound, and do the data support the conclusions?

The manuscript must describe a technically sound piece of scientific research with data that supports the conclusions. Experiments must have been conducted rigorously, with appropriate controls, replication, and sample sizes. The conclusions must be drawn appropriately based on the data presented.

Reviewer #1: Partly

Reviewer #2: Yes

2. Has the statistical analysis been performed appropriately and rigorously?

Reviewer #1: Yes

Reviewer #2: Yes

3. Have the authors made all data underlying the findings in their manuscript fully available?

Reviewer #1: No

Reviewer #2: Yes

In addition to the individual participant averages, we have uploaded two files presenting single-trials in .csv format. One table provides raw data for all 4500 trials of the experiment, the other one provides pre-processed data in percent of a participant’s individual dynamic range for all analysed trials. These files are available from the OSF repository [https://osf.io/3w5s6/].

4. Is the manuscript presented in an intelligible fashion and written in standard English?

PLOS ONE does not copyedit accepted manuscripts, so the language in submitted articles must be clear, correct, and unambiguous. Any typographical or grammatical errors should be corrected at revision, so please note any specific errors here.

Reviewer #1: Yes

Reviewer #2: Yes

5. Review Comments to the Author

**Reviewer #1:** The manuscript describes an experiment into the effects of brand-familiarity on pupil size. The experimental paradigm was designed with care to ensure that the observed pupillometric effects could be attributed to brand-familiarity rather than some other aspect of the stimuli. Brand-familiarity was initially assessed in an independent online study with a large representative sample size (n=763), precluding the requirement to obtain self-report measures from participants in the main pupillometry experiment (n=15). The study found that pupil size was greater on average when viewing familiar vs. unfamiliar brands, and additional analyses revealed how this difference is modulated by brand category and consumer behaviour.

* Is the manuscript technically sound, and do the data support the conclusions?

For the most part, the study is technically sound, but I note the following issues:
1) The sample size for the pupillometry experiment is low, especially in light of the small effect size and claims of a ‘robust indicator of brand familiarity’ (lines 492—495). Consider validating this claim by collecting more data or, alternatively, providing further statistical evidence (e.g., from simulations) that the effect is not down to chance.

Thank you for your thorough evaluation of our work. While it is undoubtedly the case that the sample size of the present study is rather small, we would like to point out that, as suggested by Smith and Little (2018), our investigation puts emphasis on differences on the individual participant level to draw our conclusion. We found, that all but one of the 15 tested participants show a clear effect of larger pupil dilation for familiar images during the second half of the viewing period, which is in the direction of the group’s average and can be seen from the single-participant plots (Figure 2c, d). The remaining participant did not exhibit a clear effect in any direction. Having 14 out of 15 participants showing a similar effect gives us confidence in our results despite the rather small sample size. Unfortunately, collecting more data is not possible at the present moment due to local restrictions necessitated by the on-going pandemic.

Nevertheless, in an effort to provide more evidence from exploratory computational modelling that our effect is not down to chance or averaging on the single-participant level, we performed a single-trial linear discriminant analysis. This analysis is commonly used in time series analysis of electrophysiological data (e.g., Diaz et al., 2017; Franzen et al., 2020; Kayser et al., 2017; Philiastides & Sajda, 2006). A model is computed that learns a weighting for optimal discrimination between data obtained from two experimental categories of interest within a moving temporal window. This trained weighting is subsequently tested on one trial that was not included in the training procedure (i.e., a leave-one-out procedure). Following signal detection theory, this test returns an area under the curve value for the ability to classify a single-trial correctly (i.e., predicting the experimental condition of this trial only using its data). In this single-participant analysis, every participant becomes its own replication unit that is explicitly tested, as suggested by Smith and Little (2018). Results show that our data affords discrimination accuracy (i.e., discriminator performance) above chance on the group level with the best performance during the last second of the viewing period (Figure A1 below). An earlier, albeit smaller, increase in discrimination performance is also visible from the plot. This finding indicates the possibility to differentiate between our familiarity conditions exclusively using the present pupil data. Hence, the familiarity difference appears to be sufficiently large to successfully classify a previously unseen single-trial as familiar or unfamiliar. This ability was present for 10 out of 15 participants. Crucially, these results are in line with the effect we observed using the cluster-based permutation approach, and thus corroborate our main finding.

We only present this analysis here, as we are of the opinion that it would be beyond the scope of this paper to include a full description of this complex analysis.

Further, please note that the bootstrap analysis implemented for the questionnaire data used the group as replication unit to extrapolate/simulate results from the available data (presented in Fig 4).

**Fig A1**. Discriminator performance group average (*n* = 13). Please note that values between 0.5 and 0.6 are common for this analysis. The analysis did not succeed for two participants.

2) The abstract indicates that the primary intention of the paper is to introduce a novel paradigm for exploring brand familiarity, which relegates the actual research to an example application of the paradigm. Note also that previous pupillometry studies have used a scrambled image mask approach (e.g., Nuske et al., 2014).

Thank you for pointing us to this paper that we had missed in our original search. While the mask used by Nuske and colleagues (2014) is similar to ours in its idea, we would like to point out that the two paradigms differ in two ways. Firstly, our scrambled mask was scrambled on the pixel level whereby larger blobs or as coherent perceived parts are entirely avoided (Fig 1b, c). Equally, using a pixel-scrambled mask precludes potential issues of the pupil dilating due to the specific location an observer gazes at during the baseline period. This effect has recently been reported and should be avoided (Derksen et al., 2018). Secondly, Nuske and colleagues used movies expressing emotions during the presentation of the actual stimuli and not still images.

**ACTION:** To honour previous applications of paradigms with a somewhat similar idea, such as the one by Nuske et al. (2014), we removed references to our paradigm being novel or innovative in itself, and clarified distinctions between earlier and our approach throughout the entire manuscript (e.g., lines 22, 97-100, 104 & 747).

3) There is limited discussion of how the observed effects may relate to established theory regarding the mechanisms of cognitive pupil control. For example, the role of the locus coeruleus, and Adaptive Gain Theory (Aston-Jones and Cohen, 2005) or ‘Network Reset’ (Bouret and Sara, 2005).

Thank you, these are relevant suggestions. In the original discussion, we avoided discussion of the physiological/neural mechanisms of cognitive pupil control, as these would have to take the form of drawing speculative links. We concur though, that a speculative, brief discussion of such mechanisms may be beneficial for the reader.

**ACTION:** In an attempt to offer some suggestions of possible physiological mechanisms underlying our results, we added the following paragraph to the discussion (lines 661-683).

“On a neural level, the pupil responses to the branded images may reflect activity of the locus coeruleus (LC), its associated connections to several brain regions, and the Adaptive Gain Theory (Aston-Jones & Cohen, 2005). The LC is a subcortical structure that is linked to the brain’s noradrenergic system and is found along the pupil dilation pathway. Cognitive processes that affect pupil dilation through the LC pathway likely involve contributions from other parts of the cortex as well (e.g., anterior cingulate cortex and the orbitofrontal cortex; (Joshi & Gold, 2020)). These two structures are associated with the concept of utility (Aston-Jones & Cohen, 2005). Under the Adaptive Gain Theory, it is suggested that the locus coeruleus-norepinephrine (LC-NE) modulatory system optimizes cognitive processes by adaptively conducting continuous evaluations of the targeted stimuli through the tonic (sustained) and phasic (event-related) modes of the LC. The phasic mode involves high task engagement and is activated in response to task-relevant events. In comparison, the tonic mode involves high distractibility and is associated with exploration (Aston-Jones & Cohen, 2005). In the current study, the results showed a greater increase in mean pupil size from baseline for familiar (vs unfamiliar) branded images while focusing on the task. This suggests that participants’ LC neurons were in phasic mode when viewing the familiar branded images, as participants might have recognized relevant content. In comparison, participants’ LC neurons may have been in tonic mode when presented with unfamiliar branded images, as the lack of familiarity may have led to greater exploration and distraction. The input signals from the anterior cingulate and the orbitofrontal cortex can generate increased activity in the phasic mode of the LC. Thus, it can be inferred that recognition of the familiar images may reflect an increase in the phasic mode of the LC, as familiar brands are thought to be more relevant to consumers (Alba & Hutchinson, 1987) and may indicate higher utility.”

4) Provide further explanation for why it is interesting to explore the pupil data with respect to brand category. What are the practical advantages of knowing this?

This research aims to bridge the fields of cognitive and consumer psychology while putting the main emphasis on the cognitive effects as reflected in pupil size changes. From a purely cognitive standpoint one could argue that information about product categories is not absolutely crucial to evidence the pupil size changes as a function of brand familiarity. Nevertheless, in consumer psychology the importance of product category has been shown as brand experience and exposure can affect consumers’ brand recall (Baumann et al., 2015). Consumers are also better able to recall a given brand if they have personally used it (Baumann et al., 2015). It can be inferred that participants’ pupil responses to the product categories may have been affected by their general usage frequency and depth of encoding of the products in each category. Hence, in considering all aspects that may give rise to product familiarity in the applied context, we also analysed brand familiarity by product category. Investigating products in applied settings, therefore, raises the question about differences between categories.

As the sample included undergraduate students, it is plausible that personal care and cleaning products were less familiar to them since they may have less experience with these product categories or pay less attention to these brands when shopping. Our group-level data underlines that consumers’ brand familiarity appears to differ somewhat by product category, as illustrated by differences in the magnitude and extent of the pupil size effect. By exploring pupil data with respect to brand category, we get insights about how brand category and potential differences in product use may affect brand familiarity.

Unfortunately, we did not collect data on the individual usage frequency of the products in the sample that took part in the pupillometry experiment. While we argue that this allows for group-level assumptions, it does not afford us to establish precise causal links between the strength of the pupil effect and the individual usage frequency on the participant level. Therefore, we present this individual participant information to the interested reader in the supplemental materials to prevent it distracting from the main aim of the manuscript itself.

**ACTION:** We added a concise version of the above argument as further motivation for the separate analysis of brand categories to the relevant paragraph of the Results section (lines 443-448).

5) Note that pupil size can be affected by illusions of brightness. Laeng et al. (2012) first showed this, but there have been replications with real-world images (e.g., Castelotti et al., 2020). The image of Ovomaltine in Figure 1c is actually redolent of the stimuli used by Laeng et al. (2012), and it is possible that brightness illusions could be affecting the data for individual stimuli. This is unlikely to pose a major issue for the study, but it is worth mentioning.

We agree with the reviewer that illusions of brightness have been shown to be able to affect pupil size. Although the stimuli used in the study by Laeng et al. (2012) and ours both feature brighter and darker spots, we consider ours to differ on two important points. Firstly, Laeng’s illusions of brightness did not feature any other informative content, with the illusionary brighter parts also being most often located in the centre of the stimulus that attention was drawn to and fixated the most. Therefore, these brighter parts were attended the most as the authors showed using heatmaps, and were considered to drive the effect. In the present study, brighter spots of a product (if any were present), were not necessarily located towards the centre of the product image, and may have not been fixated for that reason. Further, if present, these bright spots varied in size between images. Secondly, our images featured a variety of different informative elements that could have attracted the observer’s attention instead of the brighter spots. Therefore, although we cannot rule out that an illusion of brightness affected the pupil size on individual trials, we consider it unlikely that this was the crucial driver of our effects. Particularly, since the focus of our stimuli was placed on all (salient) elements of a product image, which could differ from those that carry the potential to evoke an illusion of brightness.

Lastly, the number of images that may have had the potential to lead to such illusions of brightness were somewhat equally distributed across the two conditions of interest (i.e., familiar vs unfamiliar). The exemplary product image presented in Figure 1c in the original manuscript (which had to be removed in the revised version due to copyright concerns raised by the publisher) represents one of the most colourful images in the dataset.

**ACTION:** Since we cannot entirely rule out that an illusion of brightness exerted effects on pupil size in a subset of trials, we have added a mention to the limitation section (lines 700-705).

* Has the statistical analysis been performed appropriately and rigorously?

The analyses appear to be in good order, but I have the following recommendations:
1) In addition to Maris & Oostenwald (2007), cite Sassenhagen & Draschkow (2019) and follow their recommendations for the reporting of cluster-based permutation tests. It is my understanding that these tests indicate only whether an effect was, or was not, present in the data, and that they should not be used to infer the temporal locus of an effect. For example, lines 412-416 discuss the early vs. late significant clusters, but I would have thought it would be appropriate to report only the largest significant cluster. Also specify what software was used to perform the tests (e.g., custom implementation, MNE, Fieldtrip, etc.)

Thank you for this suggestion. Sassenhagen and Draschkow (2018) make an important theoretical point about the description of the temporal locus of an effect that the field needs to be more aware of. We noticed that the description of our results would benefit from further clarification in the revised version of the manuscript. Specifically, as suggested by Sassenhagen and Draschkow, we refined all statements pertaining to specific temporal loci of the identified clusters to ensure that these statements are rather descriptive and accommodate the uncertainty in the exact temporal extent of a cluster. The authors write that it is acceptable to state approximate time points of clusters while the statement needs to point out the uncertainty regarding the specific null hypothesis of the temporal extent for instance that is inherent to such permutation tests.

While it is correct that cluster-based permutation tests show mainly the presence (or absence) of an effect compared to the general null hypothesis of the data, we implemented a slightly different percentile bootstrapping approach for dependent samples in a first step (i.e., sampling with replacement not mere shuffling of conditions). Here, the difference between familiarity conditions, obtained for a respective time point and participant, was sampled with replacement across the entire group to determine a robust 95% CI, stemming from a distribution of 1000 median (group) difference scores, for the difference at this time point. This first step already uses a non-parametric test to establish significance on a sample-by-sample basis (i.e., every 30 ms) and has been shown to be more conservative than permutation tests with sample sizes between 10 and 25 (Pernet et al., 2015), as it also allows for data substitution and not only shuffling, which affects the estimator of central tendency and the variance of a distribution.

It is only in a second step that we examined the temporal extent of significant clusters by grouping adjacent samples determined significant by the above bootstrapping procedure in a cluster and computing the size of the largest temporal cluster. Here temporal labels were permuted and the procedure repeated 1000 times. The 95^th^ percentile of the distribution of maximum cluster sizes of each of the 1000 iterations was then used as cut-off in the revised version of the manuscript. Thereby comparing the observed data to the null hypothesis of exchangeability in the temporal domain after computing results for the temporally permuted data 1000 times. This procedure resulted in a minimum cluster size threshold of 30 samples for the 3 s data, whereby the cluster we termed “early” in the original manuscript did not pass the significance test anymore.

This implementation is based on published work by Rousselet and colleagues (2016, 2017) and was obtained from the first author. Please note that we have used a similar procedure in previous work on EEG data published in Nature Communications—script available from the article’s OSF repository (Franzen et al., 2020; https://osf.io/rhx6y/).

**ACTION:** We amended the text in the Materials and Methods (lines 350-352 & 363-375) and Results (lines 387-391) sections to reflect the revised analysis and results. We also added to the Materials and Methods section that custom scripts in MATLAB were used to compute the statistical tests and the references this code was modelled on (lines 370-372).

2) Clarify why only 2.5 s of data for the 3 s stimulus period were analysed and whether this affected the significance of the results.

Thank you for giving us the opportunity to reflect on this presentation decision. Initially, we decided to present only 2.75 s of data in each plot to avoid potential lapses in attention towards the end of the viewing period to affect the main results. Given the conservative and robust statistics used, particular trimmed means and bootstrapping, a small number of outliers would not be expected to change the group-level results though. Further, this decision bears exclusive relevance for the computation of the permutation analysis threshold and the mean dilation across the entire trial, as in all other cases plots were simply terminated at 2.75 s post-stimulus onset.

Upon refining our analysis pipeline including the use of a shorter 150 ms baseline and a refined outlier thresholding procedure (for details, see response to the next point), and to be transparent to the reader, we present the entire 3 s time window in the revised manuscript. Both the original and revised analyses yield similar results including a larger pupil dilation effect in response to familiar images during the second half of the viewing period that starts around 1.4 s post image onset. Although the difference in mean pupil dilation across the entire viewing period is slightly larger for the 3 s window, the approximate start of this effect is almost identical to the results of the original manuscript. Thus, providing more evidence for the robustness of the described pupil effect towards the later stages of the viewing period. Lastly, the permutation threshold of consecutive significant samples forming a cluster is smaller for the shorter data due to shortening the late effect, however, both thresholds result in only the later effect being considered significant regardless of the extent of the analysis (2.5 s = 22 windows, 3 s = 30 windows). These thresholds represent consecutive significant differences of ~710 ms and ~950 ms, respectively.

**ACTION:** We revised the Results section to reflect our observations from the entire image viewing period of 3 s (starting in line 381).

3) It would be informative to see the pupil data for the scrambled image. Also, it would be helpful to comment on why the pupil is on average already 5% greater than baseline at the start of the stimulus period. Could this be effects of anticipation, with participants being able to accurately predict when the stimulus would appear? Note that a temporal jitter for the duration of the scrambled image may have ameliorated this affect by making it difficult for participants to predict the onset of the stimulus.

Initially, we did not mention the slight offset in the starting point of the pupil size traces explicitly, since the difference between familiarity conditions using the 500 ms baseline data did not differ significantly from zero (*t*(14) = -0.05, *p* = .9596, 95% CI = [-7.171, 6.834]; Figure B1 below). Nevertheless, a starting value of just below 5% on average is intriguing and we suspected its origins to lie in changes in pupil size over time during the baseline mask. To clarify the nature of this observation, we ran a confirmatory analysis using the last 150 ms of the baseline mask before the onset of the intact image as an alternative baseline reasoning that any changes that occur earlier during the baseline period (i.e., -500 to ‑150 ms) would still be carried over when using the median of the full 500 samples, and may result in this slight offset of ~5%. This analysis also included a computationally refined thresholding procedure for excluding trials with outliers or overly large overall pupil constriction, resulting in 117 fewer trials to be excluded (familiar = 58, unfamiliar = 59).

The results of this new analysis show that using the shorter baseline results in pupil size changes starting closer to a value of 0% on average and are virtually identical between familiarity conditions (*M*_Fam_ = 1.37%, *M*_Unfam_ = 1.31%; Fig 2a). More evidence for the efficacy of the shorter baseline in combination with computing pupil size in percent of a participant’s dynamic range accounting for inter-individual differences is provided in Fig C1 below, which demonstrates that pupil size during baseline is virtually identical between familiarity conditions using this approach. Crucially, the median difference of the two familiarity conditions during the baseline mask neither differs significantly from 0 for the longer 500 ms baseline (*Median*_500_ = 3.41, *SD*_500_ = 12.64; *t*(14) = -0.05, *p* = .9596, *g* = ‑0.0006, 95% CI_g_ = [‑0.024, 0.023]; in arbitrary units; Figure B1 below) nor for the shorter 150 ms baseline, and is almost reduced to 0 when using the shorter 150 ms baseline (*Median*_150_ = 0.61, *SD*_150_ = 12.7, *t*_150_(14) = ‑0.62, *p* = .5485, *g* = ‑0.007, 95% CI_g_ = [‑0.030, 0.016]; in arbitrary units; Fig B1 below). Hence, this speaks to a similar starting point at stimulus onset of the intact image for familiar and unfamiliar images. Please note that statistics were estimated using the median of the respective baseline of the 1000 Hz data, while the small difference visible between group averages in Figure B1 is a result of the downsampling procedure applied continuously to these data to achieve a continuous representation.

Further, it is conceivable that the slight but steady increase in pupil size during the baseline period that can be observed in Figures B1 and C1 below is a consequence of the pupillary light reflex and overall adaptation to the particular stimulus of the trial, as intended by this paradigm. This adaptation may well take up to one second. As well, we cannot rule out that effects of anticipation of stimulus onset of the intact product image have also played some role in this steady increase and acknowledge that a temporal jitter could have helped to potentially avoid such effects.

**Fig B1**. Downsampled size in arbitrary units	**Fig C1**. Downsampled size in percent

Interestingly, by using this refined analysis and the shortened baseline window the somewhat narrow early effect was reduced in temporal extent and rendered insignificant based on the temporal cluster-based permutation threshold. Conversely, the later effect remained similar in temporal locus and extent (~1400-3000ms). The number of participants showing the same effect than the group increased throughout the viewing period reaching up to 100% for the later time windows after 2 seconds. Please note, based on the arguments presented by Sassanhagen and Draschkow (2018), we have adopted a revised phrasing of this result by providing a temporal range for the starting point of this effect (i.e., 1300 to 1500 ms).

**ACTION:** As we consider the shorter baseline of 150 ms pre-stimulus onset of the intact image more representative of the final pupil size evoked by the baseline mask, we recomputed our analyses using the median value of this shorter baseline and present all results based on these new computations in the revised version of the manuscript. We now state a comparison of familiarity conditions during baseline for both the 150 ms and 500 ms baselines (lines 430-439). We have also adopted a revised phrasing of the main result that gives a reasonable temporal range for the starting point of the late effect (i.e., 1300 to 1500 ms; e.g. line 389).

4) Pupil data with EyeLink systems are affected by optical distortion due to eye movements. It would therefore help to clarify how well participants were able to maintain central fixation, and to exclude trials with large eye movements. See Hayes & Petrov (2015) for more information on this.

This is an interesting point of discussion in the field. Hayes and Petrov (2015) specify a sophisticated model to counteract the pupil foreshortening error that can lead to issues with pupil size estimation on EyeLink systems. As shown by them, this error increases with increasing visual angle from the centre of the screen. At the same time, these authors mention that keeping the gaze position relatively stable around the centre of the screen likely ameliorates the pupil foreshortening error enough for it not being considered a confound.

We did not apply this model to our data as the field is still debating whether this is the best way to correct pupil data. Other researchers have shown that using this data correction method is not the best way forward (Mathôt et al., 2018). Instead, it would be better to create a 3D model of the eye and pupil. However, we are not aware of an existing implementation for the EyeLink yet. Being aware of the pupil foreshortening error, and in combination with the absence of such a 3D model, we included only eye movements within an interest area corresponding to the actual image that was presented in the centre of the screen.

Importantly, in an effort to provide more reassuring evidence that this specific intricacy of the EyeLink system did not confound our results, we ran additional analyses computing an aggregate map of all fixations (turquoise circles and numbers) and saccades (yellow lines and numbers) associated with the trials that were included in the final analysis. As only data from one eye was analyzed for a given trial, we display the maps of fixations and saccades for both eyes separately (Figs D1 and E1 below). These maps demonstrate that most activity focused on the centre of the screen, more specifically the middle of the product image, with some occasional saccades taking place further away from the centre of the image when the data of the right eye was analyzed. These larger saccades occurred very infrequently as the right eye map includes data on ~31,000 fixations/saccades (Fig E1). Their direction and nature even suggest that some of these saccades may have even occurred immediately before or after a blink, which would have been excluded during the custom blink interpolation procedure at the pre-processing stage. In contrast, the map of fixations and saccades of the left eye displays data of ~6,500 fixations/saccades (Figs D1 and F1).

**Fig D1**. Fixation and saccades left eye	**Fig E1**. Events right eye (~31,000)

**Fig F1**. Events Left eye (~6,500 fixations/saccades)

Minor points:
1) Line 227 – It is inaccurate to say that ‘participants’ eyes’ were calibrated’, as it is the eye tracker that gets calibrated. Say rather that a 9-point calibration was performed.

Thank you for this suggestion that we have implemented in the revised manuscript (line 236)

2) Line 268 – report degrees of visual angle rather than pixels

We now report the image size in degrees of visual angle as in “subtending ~4 x 4 degrees of visual angle” (line 280).

* Have the authors made all data underlying the findings in their manuscript fully available?

Only the averages per participant are available on the OSF. Please make all of the underlying data available.

In addition to the individual participant averages, we have uploaded two files presenting single-trials. One table provides raw data for all 4500 trials of the experiment, the other one provides pre-processed data in percent of a participant’s individual dynamic range for all analysed trials. These files are available from the OSF repository [https://osf.io/3w5s6/].

* Is the manuscript presented in an intelligible fashion and written in standard English?

Note the following minor issues:
1) Line 308: Clarify what is meant by ‘pupil elasticity’

The idea of pupil elasticity, which refers to the dynamic range of an individual’s pupil size over the course of an experiment, was suggested by Winn and colleagues (2018).

**ACTION:** We have clarified this sentence and added a definition in link with the original citation (line 318-321).

2) Lines 54-56: consider revising sentence

Thank you, indeed this sentence was not fully clear.

**ACTION:** We have revised this sentence, which now reads: “This is most likely due to the difficulty of isolating the small relative change in pupil size due to cognitive processes (~1 mm) from the changes of up to 9 mm elicited by physical light/luminance (7) present in many applied settings” (lines 57-60)

3) Lines 236-237: elaborate on the claim that the background color likely avoids discomfort

A grey background colour can reduce a display’s contrast levels, and thereby eye strain and the perceived flicker that is often encountered when constantly gazing at an LCD display with high luminance and contrast. The latter can be a consequence of large proportions of pixels being shown in brighter colours including white. Most previous studies of visual discomfort have been conducted with high-contrast stimuli at photopic light levels, at which primarily only the cones operate. A recent study, using a grey screen as the baseline for a discomfort scale, demonstrated that discomfort in response to a flickering grey screen increased as luminance and contrast increased (Yoshimoto et al., 2020). This effect was mediated by the frequency of the flicker, with steady changes leading to the least discomfort. Hence, we intended to avoid increased discomfort by using a mainly grey screen similar to the background used by Yoshimoto and colleagues.

**ACTION:** We added a brief version of this argument and its citation to the Materials and Methods section (line 244-246).

4) Line 466: ‘bootrapped’

**ACTION:** Thanks for spotting this spelling mistake, which we have corrected.

5) Lines 518-520: consider revising sentence

**ACTION:** We have revised this sentence in accordance with the suggestion to tone down our language about the novelty of the presented paradigm. The revised version highlights the robustness of this different paradigm and its relevance to cognitive and consumer psychology (lines 562-565).

6) Lines 572-573: consider revising sentence

**ACTION:** We have amended the paragraphs including this sentence to reflect the revised results for product categories (lines 616-619).

**Reviewer #2: Major comments**
This paper investigates whether familiarity with images of consumer products modulates pupil responses to the onset of these images. It is found that products or brands, that are unfamiliar to observers, evoke stronger pupil constrictions around 500-1000ms and/or relatively weaker dilation at later time points than familiar products. This finding is in line with previous findings but the design is novel because the authors tested the novel/old effect on pupil size in a more applied context (i.e., market research) and tried to circumvent distorting effects of image features. The paper is well written, the study design is clear and logic, and the results are relevant. However, some of the framing and claims need to be modified before I can recommend this paper for publication. First, I expect that readers would like to know how well familiar products can be dissociated from unfamiliar products by using the pupil as an objective measure. Second, the claims concerning the proposedly innovative character of the experimental design need to be toned down.

We thank this reviewer for his time as well as positive and constructive response with nuanced suggestions that we address below.

Major point 1.
Will market research truly benefit from pupillometry? I deem the applied context of this experiment as the most novel and remarkable aspect of the study, and, as such, wonder how well the pupil dissociates familiar from unfamiliar images on a trial level. I would appreciate it if the authors could provide e.g. area under the curve measures reflecting sensitivity and specificity of pupillometry as a signal detection method. My expectation is that it will be hard to decide per product whether it is truly familiar to an observer or consumer, making pupillometry a less applicable method for market research companies. Nonetheless, perhaps the authors can provide some guidelines on whether or not and how to apply pupillometry in practice.

Taken together, the validation and eye-tracking study reported in this research provide initial evidence for the applicability and usefulness of pupillometry measures in consumer research.

In this research, the level of familiarity was assessed in terms of self-reports and pupillary response, using two samples. In the validation study, a large sample of participants completed self-report measures of brand familiarity in preparation of selection of familiar and unfamiliar brand stimuli for inclusion in the eye-tracking study. In the eye-tracking study, pupil responses to the familiar and unfamiliar brand images of a smaller sample of participants (drawn from the same population as the participants in the initial image validation study) was then measured.

In order to preclude fatigue effects due to a lengthy testing session, the eye-tracking study included measurement of pupillary responses to the brand stimuli, but did not include self-reported familiarity ratings of the stimuli. Unfortunately, the fact that the eye-tracking study did not include self-reported familiarity measures, prevents us from calculating traditional measures from traditional signal detection theory based on behavioral responses, such as sensitivity and specificity, on the individual consumer/trial level.

Nonetheless, we agree with the reviewer that a classification procedure would be of interest in the applied context of this research. In an effort to extrapolate from the current data, we performed an **exploratory analysis using a combination of** **signal detection theory and computational modelling** (i.e., a single-trial linear discriminant analysis) for providing more evidence for the paradigm’s practical relevance for marketing, and against our effect being down to chance or averaging on the single-participant level. This analysis is commonly used in time series analysis of electrophysiological data (e.g., Diaz et al., 2017; Franzen et al., 2020; Kayser et al., 2017; Philiastides & Sajda, 2006). A model is computed that learns a weighting for optimal discrimination between data obtained from two experimental categories of interest within a moving temporal window. This trained weighting is subsequently tested on one trial that was not included in the training procedure (i.e., a leave-one-out procedure). Following signal detection theory, this test returns an area under the curve value for the ability to classify a single-trial correctly (i.e., predicting the experimental condition of this trial only using its data). In this single-participant analysis, every participant becomes its own replication unit that is explicitly tested, as suggested by Smith and Little (2018). Group-level results show that our data affords discrimination accuracy (i.e., discriminator performance) above chance around two time points (~ 800 ms >2000 ms), with the best performance during the last second of the viewing period (Fig A2 below). This finding indicates the possibility to differentiate between our familiarity conditions exclusively using pupil data. Hence, the familiarity difference appears to be sufficiently large to successfully classify a previously unseen single-trial as familiar or unfamiliar. This ability, varying somewhat temporally, was present for 10 out of 15 participants. Crucially, these results are in line with the effect we observed using the cluster-based permutation approach, and thus corroborate our main finding. It further is a first indication that pupil data, when combined with an optimized paradigm, may be used to determine familiarity for single trials. This approach could gain further importance by combining it with behavioral results.

We only present this analysis here, as we are of the opinion that it would be beyond the scope of this paper to include a full description of this complex analysis.

**Fig A2**. Discriminator performance group average (*n* = 13). Please note that values between 0.5 and 0.6 are common for this analysis. The analysis did not succeed for two participants.

**Guidelines.** Thank you for this suggestion included in the revised manuscript. The value of employing pupillary measures in applied marketing research lies in the increasing accessibility and affordability of eye-trackers, and technological developments (e.g., of remote eye-trackers). Increasing ease of eye-tracker use, along with the potential reduction of study duration and participant fatigue due to reduction of self-report measures administered in testing sessions that also involve eye-tracking, enhances the appeal of pupillometry increasingly in applied research. However, this applied research is still scarce and proper guidelines would warrant an entire paper dedicated to it. Here, we attempt to provide initial guidance towards the end of the revised discussion section by speculating about the reason for the infrequent use of pupillometry, namely the lack of a flexible an easily applicable measurement paradigm, before briefly pointing out elements future research should entail and general ways forward.

**ACTION:** Your comment is nonetheless well taken, and to address it, we clarified the (primary) contribution of the current research to academic consumer research, and added a brief clarification of the steps toward the application of the measure in applied consumer psychology research, as well as the advantages of taking such an approach. You will find the following clarifications in the Discussion section of the revised manuscript (lines 717-746).

“Based on a validation study including familiarity self-reports and an eye-tracking study to obtain pupil responses, this research provides initial evidence for the validity of pupil response as a measure of brand familiarity. It contributes to consumer research in several ways. First, despite the call to supplement traditional self-report questionnaires with other measures to better capture non-conscious processes and behaviors, particularly in the domain of visual processing (Sample et al., 2020), the use of such measures is still scarce. The current research demonstrates the value of such an approach, and provides an initial multi-method triangulation of brand familiarity measures. Given that pupillometry measures can be obtained at the same time as other eye-tracking metrics, such as fixations, and saccades, this approach is also promising in terms of its efficacy in a testing environment. Second, despite the slowly increasing use of eye-tracking in consumer research—mainly in obtaining processing measures of attention—pupillometry is rarely applied, despite the recognition of its potential value in measuring consumer responses, particularly in the context of advertisements (Blackwell et al., 1970; King, 1972). This may be due to a lack of an easily applicable measurement paradigm, which the present research sought to develop and test.

Prior to application of this paradigm in applied marketing research, it would be useful to measure both self-reported brand familiarity, single-trial behavioral and pupillary responses to unfamiliar and familiar brands at the individual consumer level to demonstrate that pupil response successfully dissociates unfamiliar and familiar brands at the individual consumer and trial level. This approach would allow for the calculation of area under the curve measures to more clearly establish the sensitivity and specificity of pupillary response as a measure of brand familiarity. In addition, a company may also use a generally familiar product of the brand as a baseline for familiar stimuli. By applying the described approach, subsequent studies could then rely on pupillometry to assess brand familiarity. The value of employing pupillary measures in applied marketing research lies in the increasing accessibility and affordability of eye-trackers, and technological developments (e.g., of remote eye-trackers). Increasing ease of eye-tracker use, along with the potential reduction of study duration and participant fatigue due to reduction of self-report measures administered in testing sessions that also involve eye-tracking, enhances the appeal of pupillometry increasingly in applied research.”

Major point 2.
Currently the authors frame their paper mostly on the proposedly innovative aspect of their procedure, namely using a scrambled baseline image presentation before showing the target image. The idea is that pupil size already changes in response to the colors and luminance levels of the baseline image (see Gamlin et al., 1998, for pupil responses to colors and other features). When the target image appears thereafter, the pupil will only respond to the familiarity of the object, not to the change in feature content, which is here not of interest and adds unwanted variance to the pupil response and may potentially cover up the effect of familiarity. While I understand the argumentation, I see two problems with how this issue was addressed and “sold” to the readers. First, the scrambled images do, to some degree, but not fully control for luminance and other image features such as spatial frequency (Weiskrantz et al., 1998). For instance, brightness perception and its effects on pupil size highly depends on which regions of an image are gazed at (see Derksen et al., 2018, for subtle effects of brightness distributions across images). While the mean luminance (all pixels) of the scrambled baseline image may be equal to the luminance of the target image, the bright image borders are possibly less often fixated or covertly attended after the image is shown, still affecting the early pupil constriction and following dilation.

**Paradigm.** Thank you for pointing us to the paper by **Gamlin et al. (1998)** who indeed used a similar idea for their average luminance manipulation. Nevertheless, we see the following differences between theirs and our approach. While these authors also used pixel-scrambled disks, their colour manipulation is overlaid over the entire scrambled disk and not included in the scrambled mask. Further crucial differences are that Gamlin’s study was conducted with monkeys, and not humans, and did not use a scrambled backward mask in the later half of the experimental trial. Although we acknowledge that the general idea of a scrambled mask has been used before, the use of a colourful pixel-scrambled mask in humans and in its applied nature remains a novel aspect of the presented paradigm.

**ACTION:** In an effort to do better justice to earlier work using similar ideas for baseline manipulations of average image statistics, we clarified the similarities and differences between previous and the present paradigm better and toned down our claims throughout the manuscript as suggested. Specifically, we avoid language, such as novel, innovative or the like now. We have amended sentences about our paradigm and added a couple of clarification sentences to the Materials and Methods section as well as the Discussion of the revised version of the manuscript (e.g., lines 22, 97-100, 104 & 747). Nevertheless, we argue that the presented paradigm is novel with respect to the scrambling of a coloured image and its image content and applied context.

**Pupil size driven by location of gaze (Derksen et al., 2018).** The advantage of the presented pixel-scrambled image mask is that the spatial adjacency of pixels was fully randomized whereby each location of the baseline mask included a mix of pixels of different colours and luminance from the intact image. It follows that the location of the baseline that the observer gazed at should have not resulted in any location specific pupil effects. In line with the reviewer’s reasoning, however, it is conceivable that the initial pupil constriction seen around 500 ms after the onset of the intact image, is a consequence of overt or covert attention to the now larger coherent brighter areas (e.g., whitespace around the product). If the observer moves their eyes to the darker product just after, a pupil dilation should follow, which is the case in both familiarity conditions starting around 500 ms post image onset. Following the same logic, and assuming the local change in brightness would drive the observed effects, neither the apparent pupil dilation would continue to increase steadily until the end of the time frame nor successful single-trial classification based on pupil data alone should have been possible. A plateau or even a second decrease in pupil size would be expected if the observer gazes at brighter parts of the image—even within the product itself—in later time frames of the presentation of the intact image.

With fixation and saccades staying central throughout the viewing period, the bright background was only gazed at occasionally and/or attended covertly while the product image was being centrally explored (Figs B2, C2 and D2 below). Given the small number of those saccades and fixations shown in the figures below, effects resulting from these eye movements should average out across trials and not confound the presented results.

**Fig B2**. Fixations and saccades left eye	**Fig C2**. Events right eye (~31,000)

**Fig D2**. Events Left eye (~6,500 fixations/saccades)

Also, the current scrambled images do not adapt the pupil to spatial frequency content and the presence of an initial pupil constriction around 500ms marks that some feature changes still evoke a pupil response (spatial frequency, contrast, foveal luminance, object contours, etc.). A phase-scrambled version of an object within the images would serve as better controls (e.g., phase-scrambled images are often used in face perception studies). Second, the use of baseline images is not innovative, but a standard procedure in perception and vision sciences. The authors should thus consider toning down their claims with respect to the innovative aspect of the procedure, crediting previous work on baseline control images, and discussing the limitations of their baseline implementation.

**Spatial frequency.** Although we cannot rule out that differences in spatial frequency played some role in the present study, the robustness of our effect across images, and on the single-trial level, makes it unlikely that this would present a major confound to the presented results. While real world product packages are rather homogenous in their spatial frequency compared to the variety of stimuli in natural and applied settings— based on attempts to optimize their visual perception—these products carry a variety of spatial frequencies and are nevertheless more dynamic than gratings of one precise spatial frequency. Hence, potential differences should average out across trials of a condition, and give less importance to such an effect driving pupil size changes in the present paradigm.

**Phase manipulations** of objects in images, as is frequently employed in studies investigating faces, is another reasonable option with respect to the control of low-level image statistics indeed. One of the authors has used this technique in an audio-visual face perceptual decision-making study recently (Franzen et al., 2020). However, we decided against using phase manipulation for three reasons. Firstly, the size of product packages and their contours carry a lot of information that could have been hinted at more if we had used a phase manipulation technique—also giving crucial importance to the exact percentage of phase coherence/scrambling being used. It was our intention not to give away any clue about the products to be displayed products other than their colour spectrum represented by individual pixels. Secondly, while phase manipulations can make it impossible to identify an object reliably, it preserves larger areas within an image that may appear like a coherent object, an illusionary brighter area or even a source of light due to top-down effects (Castellotti et al., 2020; Laeng & Endestad, 2012; see Fig E2 below). Thirdly, we consider phase manipulations of images (i.e., imaginary numbers) an advanced concept that may not be easily accessible to marketers and a wider audience. Thus, it could have distracted from the applied nature and applicability of this study, as pointed out in the first comment by this reviewer. These effects are illustrated in the spectrum of phase coherence illustrated for one exemplary product image below (Fig E2).

Full phase randomization will remove the higher order image statistics but will not impact other aspects of the image properties (Loschky et al., 2010). In particular, the amplitude spectrum (amplitude plotted across spatial frequency) of a phase randomized image is sufficient to allow for scene gist (outlined in Oliva and Torralba’s (2001) spatial envelope computational model which was 86% accurate on classifying an image based on the spectrum alone) and scene categorization (Guyader et al., 2005). This also becomes problematic in that unlike other natural stimuli (e.g., natural scenes, faces), products often contain high amplitudes of horizontal and vertical information from the packaging. This results in an over-representation of amplitude in these orientations that phase randomization cannot overcome (see example below).

**ACTION:** As phase manipulations are one alternative to render the content of an image incoherent without altering crucial image statistics, we briefly discuss this technique (lines 705-716).

**Fig E2.** Examples of phase coherence manipulations between 0 and 100% in steps of 10% for an exemplary face image (top row; Face Database MPI Tübingen (Troje & Bülthoff, 1996)) and for a grayscale version of a product image used in the current study (bottom row).

Minor comments
- 69-70 it will help the reader to clarify what is meant with implicit indicators. Do you mean measures from tasks like the implicit association task (IAT)?

We would not consider the implicit association task as one of the measures to determine implicit indicators of brand familiarity. Although we have attitude objects that can hold implicit associations, with regards to brand familiarity, only one of these objects could be linked to an evaluative attribute (i.e., with familiar objects since unfamiliar objects would be ad hoc-generated).

**ACTION:** We have clarified that we mean physiological responses that are not governed by conscious control by implicit indicators in this part of the Introduction (line 72-73).

- Please double-check the references (e.g. Sirois & Brisson misses the title).

The title of this publication is simply “Pupillometry” and was already present in the original version of the manuscript. We acknowledge, however, that this very brief title may easily appear like a missing title when using Vancouver style.

References
Derksen, M., van Alphen, J., Schaap, S., Mathot, S., & Naber, M. (2018). Pupil mimicry is the result of brightness perception of the iris and pupil. Journal of cognition, 1(1).
Weiskrantz, L., Cowey, A., & Le Mare, C. (1998). Learning from the pupil: a spatial visual channel in the absence of V1 in monkey and human. Brain: a journal of neurology, 121(6), 1065-1072.
Gamlin, P. D., Zhang, H., Harlow, A., & Barbur, J. L. (1998). Pupil responses to stimulus color, structure and light flux increments in the rhesus monkey. Vision research, 38(21), 3353-3358.

Signed review
Marnix Naber

**References**

Alba, J. W., & Hutchinson, J. W. (1987). Dimensions of Consumer Expertise. *Journal of Consumer Research*, *13*(March).

Aston-Jones, G., & Cohen, J. D. (2005). An integrative theory of locus coeruleus-norepinephrine function: Adaptive gain and optimal performance. *Annual Review of Neuroscience*, *28*, 403–450. https://doi.org/10.1146/annurev.neuro.28.061604.135709

Baumann, C., Hamin, H., & Chong, A. (2015). The role of brand exposure and experience on brand recall-Product durables vis-à-vis FMCG. *Journal of Retailing and Consumer Services*, *23*, 21–31. https://doi.org/10.1016/j.jretconser.2014.11.003

Blackwell, R. D., Hensel, J. S., & Sternthal, B. (1970). Pupil dilation: what does it measure? *Journal of Advertising Research*, *10*(4), 15–18.

Derksen, M., van Alphen, J., Schaap, S., Mathot, S., & Naber, M. (2018). Pupil Mimicry is the Result of Brightness Perception of the Iris and Pupil. *Journal of Cognition*, *1*(1), 1–16. https://doi.org/10.5334/joc.34

Diaz, J. A., Queirazza, F., & Philiastides, M. G. (2017). Perceptual learning alters post-sensory processing in human decision-making. *Nature Human Behaviour*, *1*, 1–9. https://doi.org/10.1038/s41562-016-0035

Franzen, L., Delis, I., Sousa, G. De, Kayser, C., & Philiastides, M. G. (2020). Auditory information enhances post-sensory visual evidence during rapid multisensory decision-making. *Nature Communications*, *11*, 5440. https://doi.org/10.1038/s41467-020-19306-7

Guyader, N., Chauvin, A., Bert, L., Mermillod, M., Hérault, J., & Marendaz, C. (2005). Rapid visual scene categorization relies mainly on amplitude spectrum. *Investigative Ophthalmology & Visual Science*, *46*(13), 5642.

Joshi, S., & Gold, J. I. (2020). Pupil Size as a Window on Neural Substrates of Cognition. *Trends in Cognitive Sciences*, *24*(6), 466–480. https://doi.org/10.1016/j.tics.2020.03.005

Kayser, S. J., Philiastides, M. G., & Kayser, C. (2017). Sounds facilitate visual motion discrimination via the enhancement of late occipital visual representations. *NeuroImage*, *148*(January), 31–41. https://doi.org/10.1016/j.neuroimage.2017.01.010

King, A. S. (1972). Pupil size, eye direction, and message appeal: Some preliminary findings. *Journal of Marketing*, *36*(3), 55–58.

Loschky, L., Hansen, B. C., Sethi, A., & Pydimarri, T. N. (2010). The role of higher order image statistics in masking scene gist recognition. *Attention, Perception & Psychophysics*, *72*(2), 427–444. https://doi.org/10.3758/APP.72.2.427

Mathôt, S., Fabius, J., Van Heusden, E., & Van der Stigchel, S. (2018). Safe and sensible preprocessing and baseline correction of pupil-size data. *Behavior Research Methods*, *50*(1), 94–106. https://doi.org/10.3758/s13428-017-1007-2

Oliva, A., & Torralba, A. (2001). Modeling the shape of the scene: A holistic representation of the spatial envelope. *International Journal of Computer Vision*, *42*(3), 145–175. https://doi.org/10.1023/A:1011139631724

Pernet, C. R., Latinus, M., Nichols, T. E., & Rousselet, G. A. (2015). Cluster-based computational methods for mass univariate analyses of event-related brain potentials/fields: A simulation study. *Journal of Neuroscience Methods*, *250*, 85–93. https://doi.org/10.1016/j.jneumeth.2014.08.003

Philiastides, M. G., & Sajda, P. (2006). Temporal characterization of the neural correlates of perceptual decision making in the human brain. *Cerebral Cortex*, *16*(4), 509–518. https://doi.org/10.1093/cercor/bhi130

Rousselet, G. A., Foxe, J. J., & Bolam, J. P. (2016). A few simple steps to improve the description of group results in neuroscience. *European Journal of Neuroscience*, *44*(9), 2647–2651. https://doi.org/10.1111/ejn.13400

Rousselet, G. A., Pernet, C. R., & Wilcox, R. R. (2017). Beyond differences in means: Robust graphical methods to compare two groups in neuroscience. *European Journal of Neuroscience*, *46*(2), 1738–1748. https://doi.org/10.1111/ejn.13610

Sample, K. L., Hagtvedt, H., & Brasel, S. A. (2020). Components of visual perception in marketing contexts: a conceptual framework and review. *Journal of the Academy of Marketing Science*, *48*(3), 405–421. https://doi.org/10.1007/s11747-019-00684-4

Smith, P. L., & Little, D. R. (2018). Small is beautiful: In defense of the small-N design. *Psychonomic Bulletin & Review*, *25*(6), 2083–2101.

Troje, N. F., & Bülthoff, H. H. (1996). Face recognition under varying poses: The role of texture and shape. *Vision Research*, *36*(12), 1761–1771. https://doi.org/10.1016/0042-6989(95)00230-8

Winn, M. B., Wendt, D., Koelewijn, T., & Kuchinsky, S. E. (2018). Best Practices and Advice for Using Pupillometry to Measure Listening Effort: An Introduction for Those Who Want to Get Started. *Trends in Hearing*, *22*, 1–32. https://doi.org/10.1177/2331216518800869

Yoshimoto, S., Jiang, F., Takeuchi, T., Wilkins, A. J., & Webster, M. A. (2020). Visual discomfort from flicker: Effects of mean light level and contrast. *Vision Research*, *173*(May), 50–60. https://doi.org/10.1016/j.visres.2020.05.002
